# Supplementary figures and images for: Exome sequencing identifies novel genetic variants associated with varicose veins
Source: PLoS Genet. 2024 Jul 9;20(7):e1011339. doi: 10.1371/journal.pgen.1011339 (PMC11233024; doi:10.1371/journal.pgen.1011339)

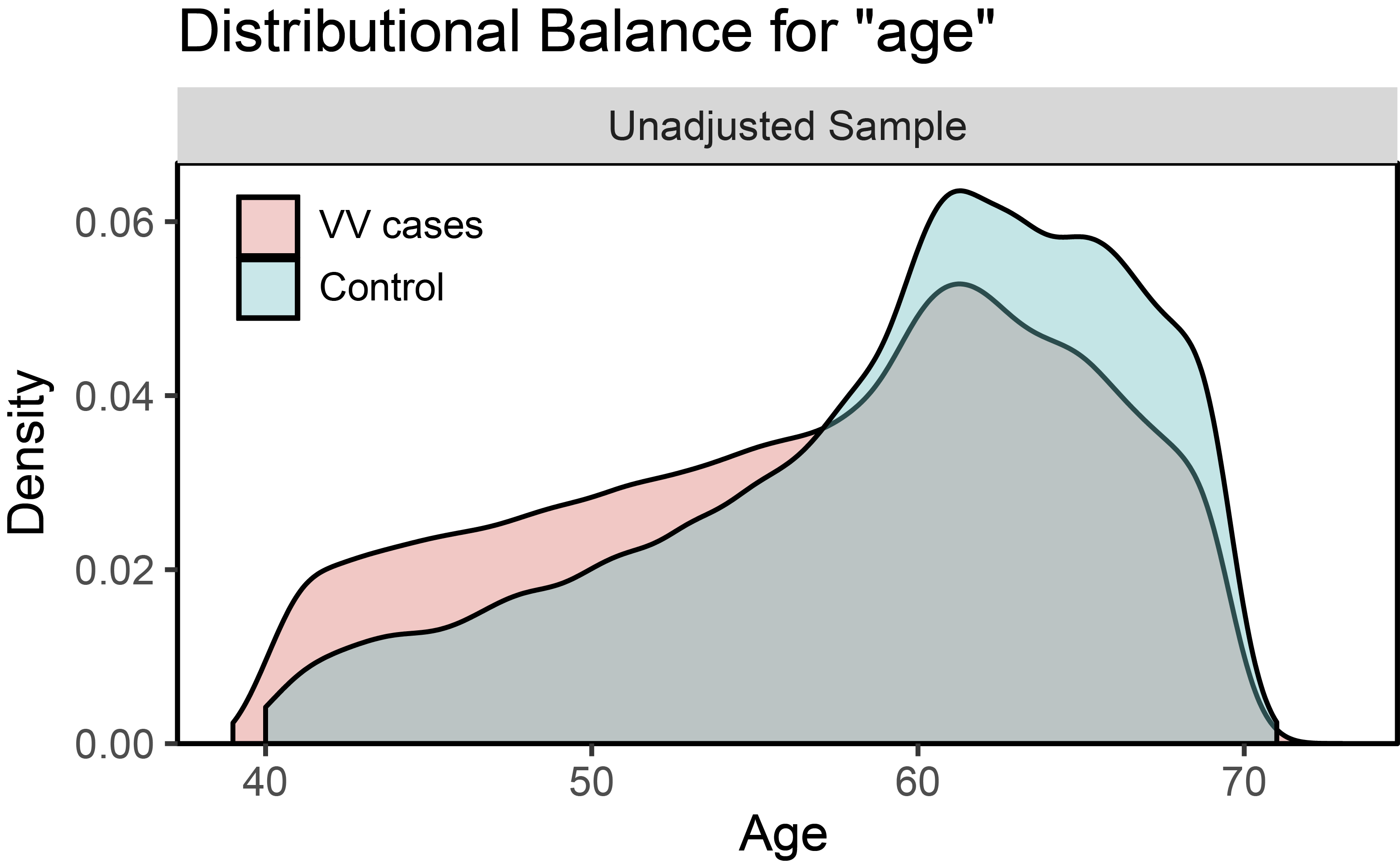

Supplement: S1 Fig — Abbreviation: VV, varicose veins. (TIF) [file pgen.1011339.s001.tif]

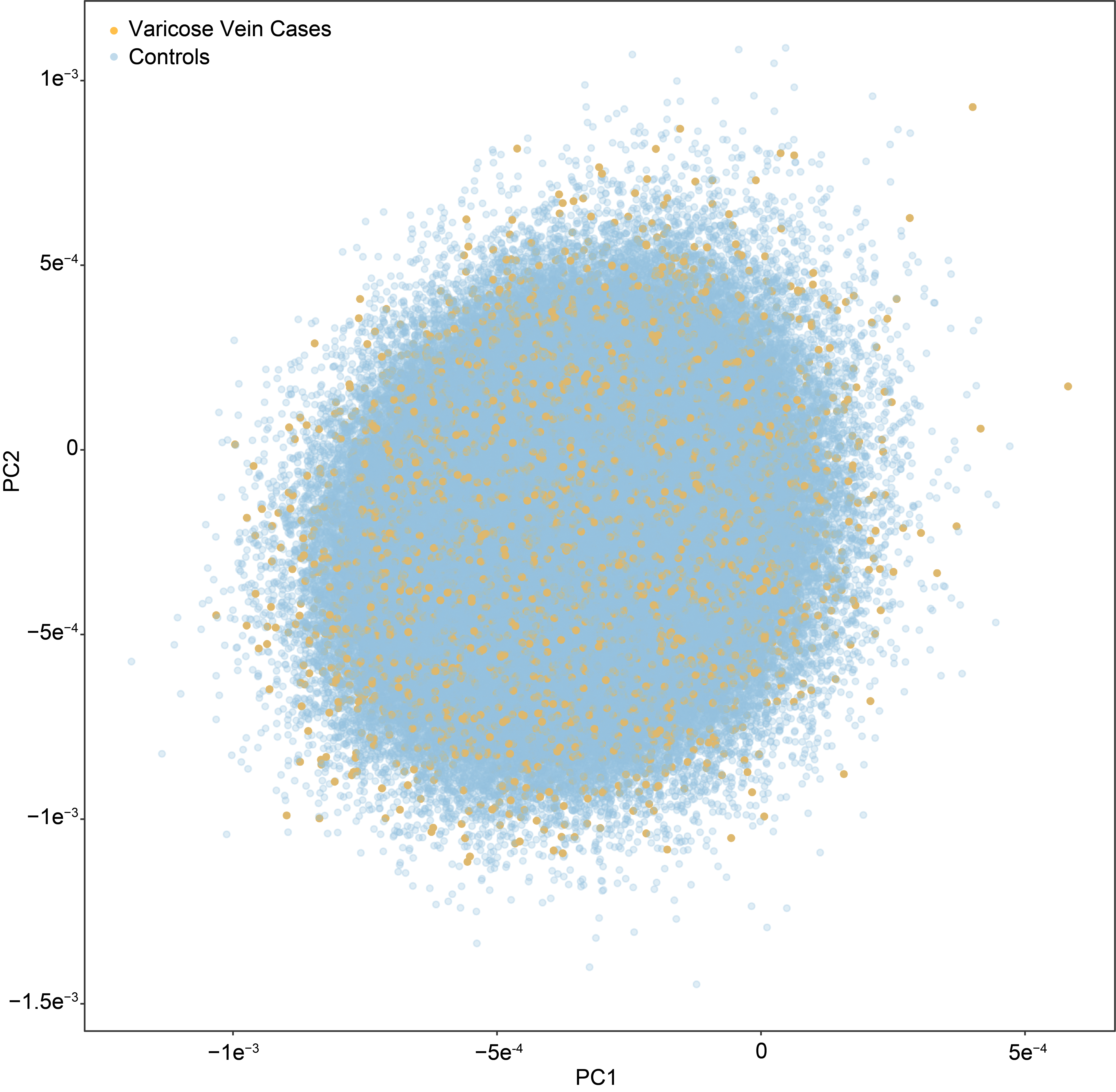

Supplement: S2 Fig — The x-axis and y-axis represent the values of the two components of PCA (PC1, PC2), and each point in the figure represents an individual. Abbreviation: VV, varicose veins; PC, principle components. (TIF) [file pgen.1011339.s002.tif]

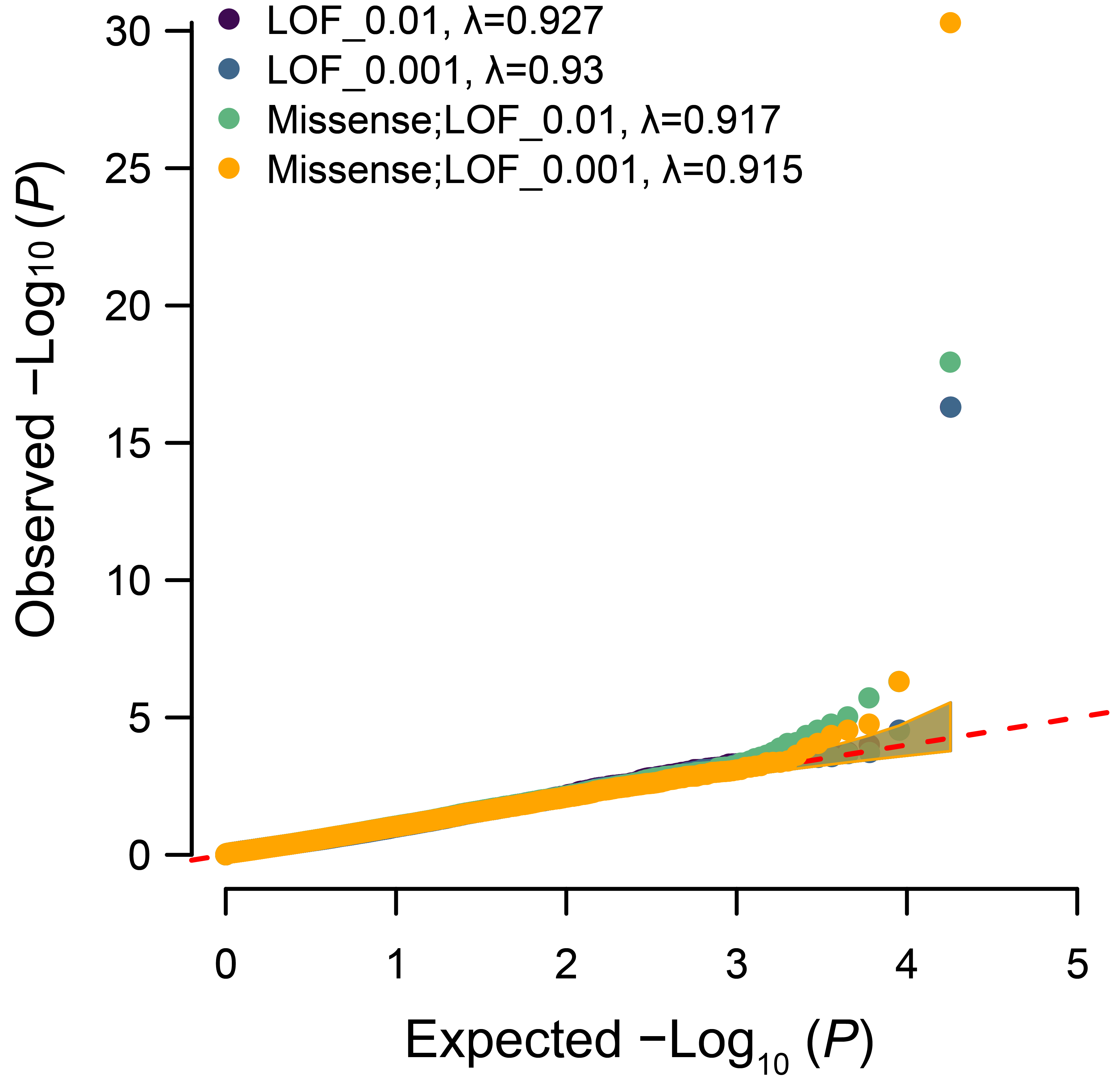

Supplement: S3 Fig — The y-axis represents the observed–log10 P values across all tests, while the x-axis represents the expected under the null-hypothesis. P values were obtained from the results of the gene-based collapse tests for varicose veins, using SAIGE-GENE+ software. In the gene-based collapse test, we applied two different maximum MAF cutoffs (0.01 and 0.001) and two different variant annotation groups (LOF and LOF + missense) to perform burden tests. All models were adjusted for age, sex, and top ten principal components. Abbreviation: VV, varicose veins; LOF, loss of function; λ, lambda. (TIF) [file pgen.1011339.s003.tif]

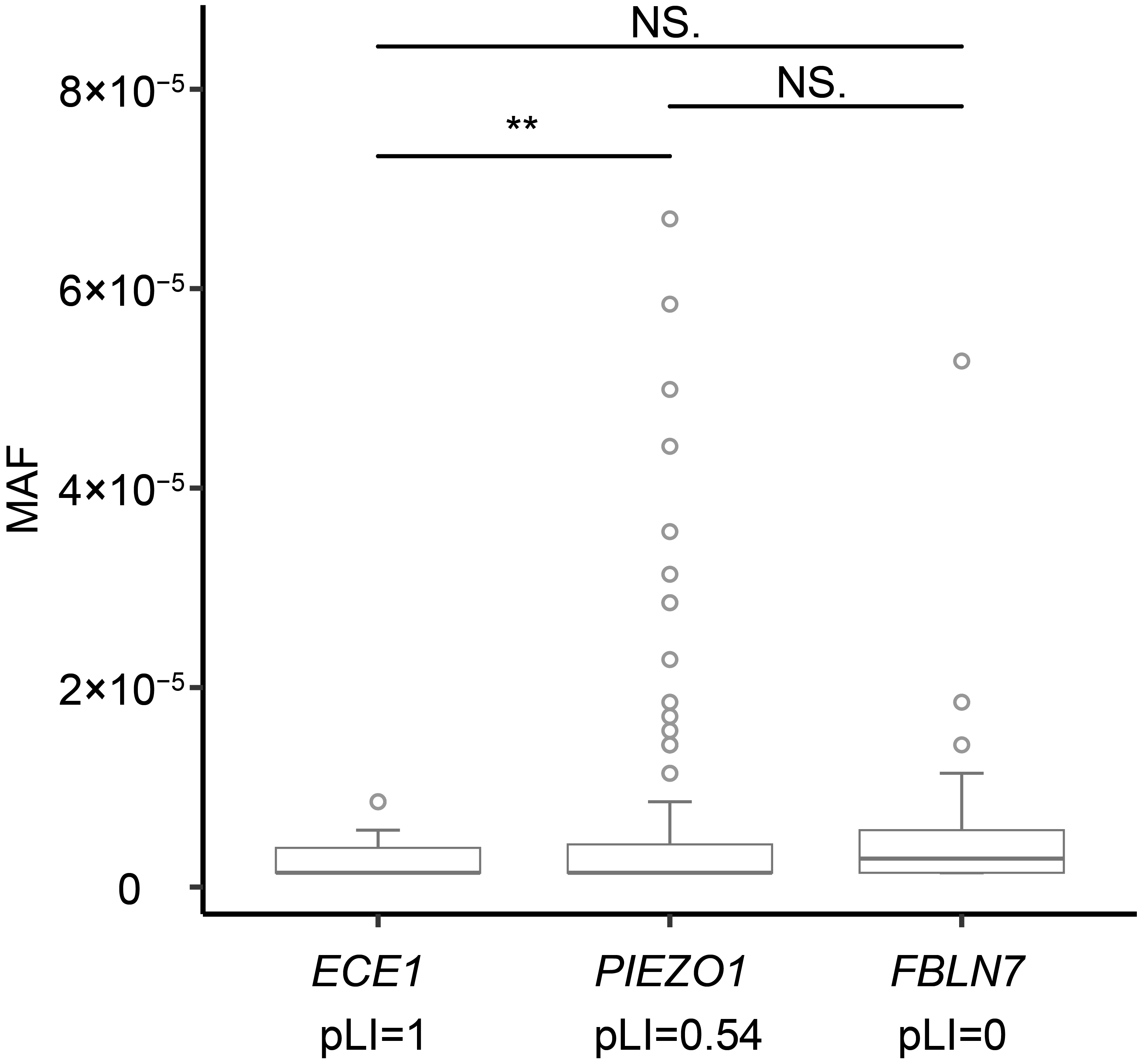

Supplement: S4 Fig — P-values for differences between groups were calculated by t-test. MAF, minor allele frequency, NS., no significance, pLI, the probability of intolerance to loss of function. (TIF) [file pgen.1011339.s004.tif]

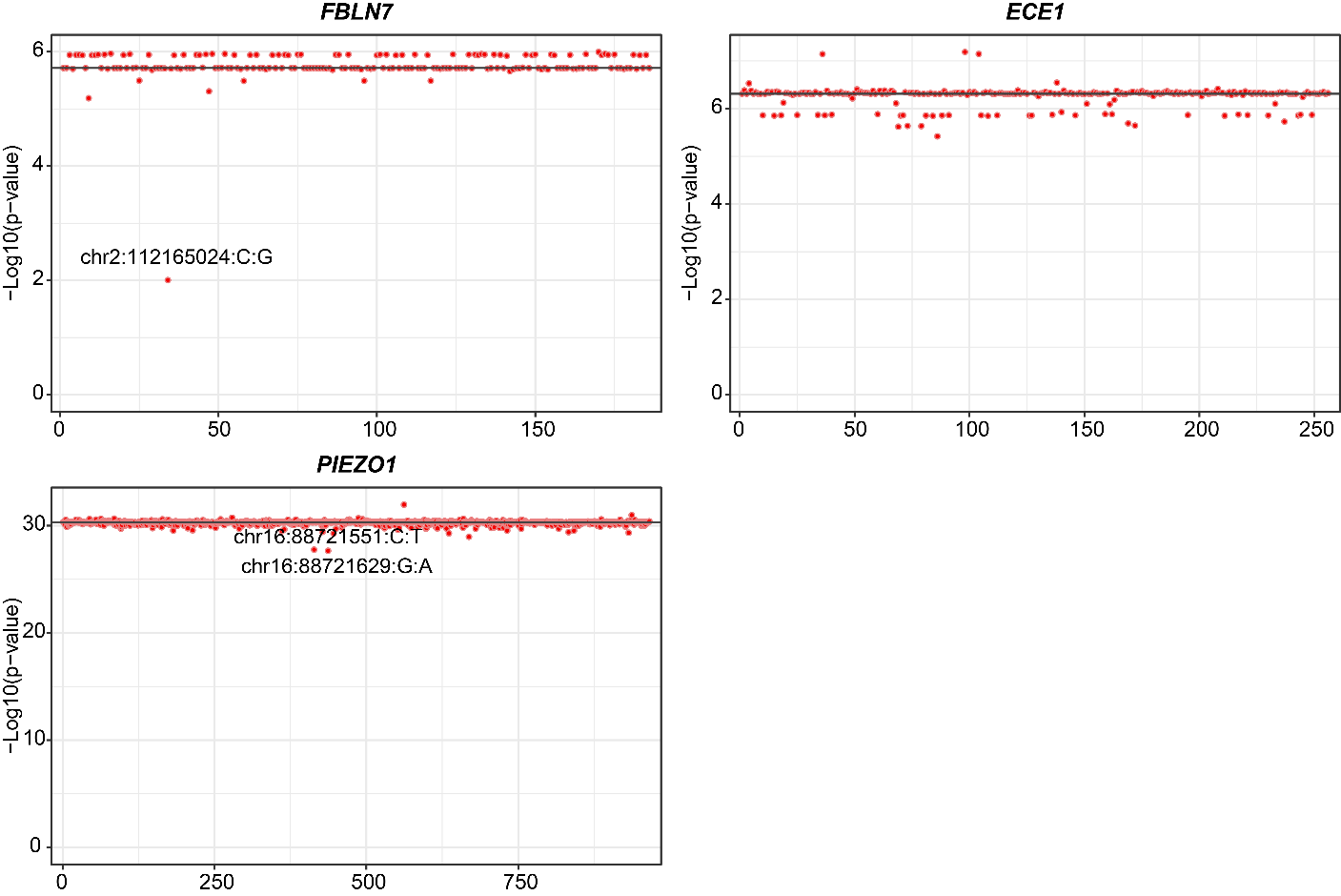

Supplement: S5 Fig — The x-axis indicates variants removed from the gene-based collapsed test, and the y-axis indicates the -log10 P-value for associations without that variant P-values were derived from the gene-based collapsed test using the SAIGE-GENE+ software, adjusted for age, sex, and the top 10 principal components. (TIF) [file pgen.1011339.s005.tif]
