# Supplementary material for: Exome sequencing identifies novel genetic variants associated with varicose veins
Source: PLoS Genet. 2024 Jul 9;20(7):e1011339. doi: 10.1371/journal.pgen.1011339 (PMC11233024; doi:10.1371/journal.pgen.1011339)
Supplement: S1 Text — (DOCX) [file pgen.1011339.s006.docx]

**S1 Text**

**Supplementary Methods**

## The definitions of deleterious variants for each software

The cut-off used in SIFT(1): Positions with normalized probabilities less than 0.05 are predicted to be deleterious, and those greater than or equal to 0.05 are predicted to be tolerated.

The cut-off used in PolyPhen-2(2): Current version 2.2 of the PolyPhen-2 uses 5%/10% false positive rate (FPR) for HumDiv model and 10%/20% FPR for HumVar model as the thresholds for this ternary classification. Mutations with their posterior probability scores associated with estimated false positive rates at or below the first (lower) FPR value are predicted to be probably damaging (more confident prediction). Mutations with the posterior probabilities associated with false positive rates at or below the second (higher) FPR value are predicted to be possibly damaging (less confident prediction). Mutations with estimated false positive rates above the second (higher) FPR value are classified as benign.

The cut-off used in LRT (Likelihood Ratio Test)(3): Deleterious mutations were predicted by nonsynonymous SNPs that disrupt significantly constrained codons defined by the LRT (P < 0.001) and several subsequent filters.

The cut-off used in MutationTaster(4): If a variant is marked as probable-pathogenic or pathogenic in ClinVar(5), it is automatically predicted to be disease-causing, i.e. disease-causing automatic (the naive Bayes classifier is run nevertheless and the p-value for the prediction is shown).

**References**

1. Ng PC, Henikoff S. SIFT: Predicting amino acid changes that affect protein function. Nucleic Acids Res. 2003;31(13):3812-4.

2. Adzhubei I, Jordan DM, Sunyaev SR. Predicting functional effect of human missense mutations using PolyPhen-2. Current protocols in human genetics. 2013;Chapter 7:Unit7.20.

3. Chun S, Fay JC. Identification of deleterious mutations within three human genomes. Genome research. 2009;19(9):1553-61.

4. Schwarz JM, Cooper DN, Schuelke M, Seelow D. MutationTaster2: mutation prediction for the deep-sequencing age. Nature methods. 2014;11(4):361-2.

5. Landrum MJ, Lee JM, Riley GR, Jang W, Rubinstein WS, Church DM, et al. ClinVar: public archive of relationships among sequence variation and human phenotype. Nucleic Acids Research. 2013;42(D1):D980-D5.
