# Supplementary material for: Exome sequencing identifies novel genetic variants associated with varicose veins
Source: PLoS Genet. 2024 Jul 9;20(7):e1011339. doi: 10.1371/journal.pgen.1011339 (PMC11233024; doi:10.1371/journal.pgen.1011339)
Supplement: Attachment — Submitted filename: Review_PlosG_2023.docx [file pgen.1011339.s027.docx]

In this study, Zhang et al. investigate the genetic architecture of varicose veins (VV) through the analysis of whole-exome sequencing data in the UK Biobank. Overall, the authors describe a substantial number of new associations with several genes, both from common and rare variant analyses. Coupled with gene set enrichment and expression analyses, the authors provide useful biological interpretation of these findings which help in better understand the disease. However, I have some comments on the manuscript before it can be accepted to publication.

**Major comments**

This study is not the first association study performing rare variant burden analyses. Van Hout et al. have indeed investigated associations between rare variants and multiple phenotypes, including varicose veins, through LoF burden analyses (10.1038/s41586-020-2853-0). The authors should at least compare their results to this study.

Age being an important risk factor of the disease, it would be useful to have the age distribution in cases and controls in addition of the overall age statistics given in l. 113. If there is a large difference between cases and controls and age is included as a covariate, then it could mask some signals. If this is the case, I would suggest performing a sensitivity analysis with a selection of controls to have a similar age distribution as the cases as the number of controls shouldn’t be an issue in the UK Biobank.

Why is the sex-specific analysis only performed with the rare variants and not with the common ones using single-point associations? It would be interesting to see whether additional variants come up when studying the common variants and whether there is also a signal in *METTL21A*.

Some clarifications on the methods used are needed:

- For the common single-point associations, the significance threshold was computed based on the Bonferroni correction, but was the correlation between the variants considered to compute this threshold? It would also be interesting to report the number of signals significant at the genome-wide significance threshold. Actually, most of the signals that are not replicated in the FinGen cohort are not significant at the genome-wide level.
- What significance threshold was used for the replication cohort?
- Can the authors give more details about the collapsing analyses? Which burden score and which software were used to perform these analyses? In addition, it would be useful in table 2 to have the number of variants retained in each testing unit to compare the results of the different masks.
- l.211: on which set of genes the gene enrichment analysis was performed?
- For the PheWAS analysis, what exactly is tested? Does it focus on single-variant associations? Also, what is the exact significance threshold? There is a discrepancy between the methods and the figures. I guess there is a typo, at least in Fig. 4 where it is stated that the threshold is corrected for 3 genes while more than 3 genes are analyzed.
- Can the authors explain in a little bit more details how they computed the disease prevalence for each variant? How did they combine LoF and missense variants?
- More details on the Gene-SCOUT analysis would also help the reader.

There are some sentences in the text that I not fully agree with or that would need additional clarification:

- l.96: how rare variants can help in evaluating disease prevalence?
- l.88: how rare variants can more directly inform causal genes? At the end, GWAS and collapsing tests are both association tests, the difference being in how the variants are analyzed
- I think there is a confusion between the effect sizes and the p-values. Indeed, it says at l. 151 that effect sizes are enhanced by the inclusion of missense variants. This is not true when looking at table 2 where p-values are indeed lower when missense are included, but the effect sizes (OR) are smaller. Similarly, in lines 204-205, the authors mention the different effect sizes but report only p-values and not OR.
- What do the authors mean by “genetic variants identified in population-based whole exome-sequencing studies were more reliable” (l. 255)? It has been actually showed that rare variant analyses are prone to more biased compared to common variants.
- l.319: I wouldn’t say that the overlapping genes are more accurate, it actually depends on the genetic architecture of the trait. It is possible that some genes only present common variation that is associated to the disease.

Overall, I would suggest the authors to carefully proofread the manuscript and improve the writing to make things clearer.

**Minor comments:**

- The single common variant analysis seems a little bit inflated. What is the lambda value?
- Why only four masks out of the 8 are reported in table 2?
- L.134-135: I don’t agree with the sentence that this is especially true for novel signals, we don’t see a large difference in Fig.1 in effect sizes and frequency distributions between novel and previous variants.
- Would the genes still be significant in collapsing analyses if the significance threshold is corrected by the number of masks used?
- In Fig. 3, we see a very distinct signature for some genes such as *SEPRRIND1* and *SLC17A2* in the liver for example. Is this something known for this gene or something that could be linked to the disease?
- l.231: what are the numbers in parentheses?
- Why is the past tense used in multiple sentences? (l. 283, l.295. l.303 for example)
- l.404: could the authors show the PCA in a supplementary figure with cases and controls labelled?
- There are some typos in the text. For example: Keywords: casual genes; l.331: “used”; l. 446: MAF of each variant, not of each gene; l.299: “to treat”
