# Supplementary material for: Exome sequencing identifies novel genetic variants associated with varicose veins
Source: PLoS Genet. 2024 Jul 9;20(7):e1011339. doi: 10.1371/journal.pgen.1011339 (PMC11233024; doi:10.1371/journal.pgen.1011339)
Supplement: Attachment — Submitted filename: Response letter.docx [file pgen.1011339.s028.docx]

The authors sincerely appreciate the critical reviews of the paper, and for the helpful way in which the reviewing editors put together a constructive list of suggestions for the revision of the paper. We have now revised the paper to carefully address all the points raised. Our responses below are preceded by “-”, and changes made to the paper are shown below within “…”, and in red font in the revised paper.

**Detailed Response to Editor**

- Many thanks for your positive feedback and for your high concern towards our manuscript. The reviewers' valuable comments indeed provided important guidance to our research. By thoughtfully integrating their insights, we revised the paper and believe the manuscript's quality has been improved.

**Detailed Response to Reviewers**

**Response to Reviewer #1**

Reviewer #1:

In this manuscript the authors report their findings following an association study into the genetic factors leading to the individuals to manifest varicose veins with the aim of providing novel target genes, strengthening the evidence of existing associated genes and hopefully provide new directions in treatment development. The authors present an overwhelming set of data in support of theirs findings for an association between genetic variants in genes and VV, although the authors present strong significant association between a number of genes the odds ratios presented are predominantly quite close to 1 and within the confidence intervals (particularly the PIEZO1 findings) suggesting that the likely association is a bit more moderate than what the authors are claiming even within this larger population dataset. I recommend that the authors consider revising their interpretation of strength of the associations detected in their study while addressing the comments below.

Major comments

1) The authors conclude that both PNO1 and PIEZO1 are strongly associated with VV and while their results and interpretation for the PIEZO1 is clearly presented in depth in this manuscript the PNO1 is very briefly mentioned at the start of the results section. I strongly recommend that the authors expand their presentation of the results that support the PNO1 association with VV which can only strengthen their conclusions.

**Response:**

- Thank you for your kind comments. We have highlighted the *PON1* in the **Discussion** section, adding a possible explanation for the association between *PON1* and VV. We added that “*PNO1 is predominantly expressed in the liver and spleen, and slightly in the thymus, testis, and ovary, but not in the heart or brain(1). In addition, it has been suggested that PNO1 may be involved in the expression of calmodulin phosphatase, a key signaling component in angiogenesis, and that calmodulin phosphatase activity is essential for vascular development(2, 3)*”.
- **Reference**:

1.	Zhou GJ, Zhang Y, Wang J, Guo JH, Ni J, Zhong ZM, et al. Cloning and characterization of a novel human RNA binding protein gene PNO1. DNA sequence: the journal of DNA sequencing and mapping. 2004;15(3):219-24.

2.	Fukaya E, Flores AM, Lindholm D, Gustafsson S, Zanetti D, Ingelsson E, et al. Clinical and Genetic Determinants of Varicose Veins. Circulation. 2018;138(25):2869-80.

3.	Graef IA, Chen F, Chen L, Kuo A, Crabtree GR. Signals transduced by Ca(2+)/calcineurin and NFATc3/c4 pattern the developing vasculature. Cell. 2001;105(7):863-75.

2) On a similar track to the previous comment, the authors should consider presenting their findings for the novel association of TRIM10 and UBE2H in a manner that supports their statements in the conclusion (lines 271 to 276). This section of the conclusion would also benefit from a restructuring of the text to clearly state the novelty of the association of these genes with VV, something that is not immediately clear from the text as is.

**Response:**

- Thank you for your kind comments. We supplemented a presentation of the association results for *TRIM10* and *UBE2H* in the **Results** (“*Then, the most strongly newly identified gene associated with VV was TRIM10 (P = 5.29 × 10^-14^)*” and “*It was found that several VV genes were significantly enriched in protein K48-linked ubiquitination (P = 7.20 × 10^-3^), including UBE2H, TRIM31, and RNF5*”).

3)The statement in the conclusion line 303-303 that the "study demonstrated the association of these genes with endothelial cells and some inflammatory markers..." requires that the authors to clearly elaborate and present this association for the 3 genes not just for the PIEZO1. The authors provide the supporting evidence in additional files, although this is not clear enough to allow a reader of this manuscript to follow the evidence to the author's conclusions.

**Response:**

- Thank you for your kind comments. We decided to delete this ambiguous sentence after combining it with the previous presentation of the **Results**.

4) The authors state that one of the strengths of their study is that this "study is the first exome-wide association study of VVs based on a large-scale population". Can the authors claim this as the first such study when other studies like the ones cited by the others in this paper have used the UK Biobank data for association studies with VV and the authors are using a collapsed subset of the data in the UK Biobank. It might be a semantics argument, but it has relevance in a precise scientific unbiased context.

**Response:**

- Thank you for your kind comments. According to your suggestions, we have changed the statement in the **Discussion** “study is the first exome-wide association study of VV based on a large-scale population” to “*Our study is the largest sample size of VV-related whole-exome association studies to date*”.

5a) Line 34 Abstract/Line 62 Author Summary/Line102 Introduction - The authors state "...most genes were validated in FinnGen." Can the authors provide more precise information about the level of validation performed in this study here? Also, should it not read "...most variants were validated in FinnGen", my understanding is that FinnGen validates the association of genetic variations with a variety of diseases.

**Response:**

- Thank you for your kind comments. According to your suggestions, we have changed the statement “...most genes were validated in FinnGen” to “..*.most associations between genes and VV were validated in FinnGen*” in **Abstract** and in **Author Summary**. We also replaced the sentence “We identified 19 known and 17 novel causal genes, and most of them were validated in FinnGen” to “*We identified 19 known and 17 novel causal genes significantly associated with VV, with most of these associations validated in FinnGen*” in the **Introduction**.

5b) The validation results obtained from FinnGen by the authors should be presented clearly in the main manuscript as evidence to support their claims and not be limited to additional supporting documents and remotely accessible material. If the authors are confident that this validation strengthens their findings it should be presented accordingly. This will also allow the authors to better address the above related comment 5a)

**Response:**

- Thank you for your kind comments. We presented *P*-values for these VV-related genes validated in FinnGen in **Tables 1 and 2**. Then we have changed the results of the FinnGen validation to be more specific by replacing “Thirty-two genes were successfully validated in FinnGen” with “*The association between 32 genes and VV was successfully validated in FinnGen*”, and “The three genes identified in our collapse analysis were further successfully validated in FinnGen” with “*The association of the three genes we identified in the collapse analysis with VV was further successfully validated in the VV genomic data from FinnGen (P ranged from 2.39 × 10^-59^ to 4.93 × 10^-5^,* ***Table 2****)*”.

6) As a general comment to the association of LoF variants in the PIEZO1 gene, have the authors considered the predicted loss-of-function tolerance (pLI=0) available from gnomAD and the recessive manner this gene is predicted to act in their interpretation of the association of genetic variation in this PIEZO1 with VV. Together with the predicted highly intolerant to LoF (pLI=0.99) of the ECE1 gene and loss-of-function tolerance (pLI=0) of FBLN7 this may help explain the carriers percentages presented in figure 2b.

**Response:**

- Thank you for your kind comments. We have extracted the pLI scores (the probability of being LOF intolerant)(4) for the significant genes. Through comparative analysis of variant carrier frequencies across the three genes (utilizing inter-group t-tests), we observed significant differences in the MAF of variants in *ECE1* (pLI = 1) and *PIEZO1* (pLI = 0.54), indicating a discernible correlation between higher pLI scores and variant prevalence (**Additional file 3, Fig. S3**). We have added it in “**Results - Three VV genes identified by collapse analysis**” (“*PIEZO1 had the highest carrier frequency (3.77%, pLI = 0.54), followed by FBLN7 (1.47%, pLI = 0) and ECE1 (0.21%, pLI = 1) (Fig. 2b, Additional files 7 and 8).* *The high pLI of the ECE1 gene underscores its pronounced intolerance to LoF variants, aligning with our findings of reduced carrier frequency (****Additional file 3, Supplementary Fig. S3****)*”.
- **Response**:

4.	Lek M, Karczewski KJ, Minikel EV, Samocha KE, Banks E, Fennell T, et al. Analysis of protein-coding genetic variation in 60,706 humans. Nature. 2016;536(7616):285-91.

7)In Figure 3 the authors present RNAseq data and expression analysis results from datasets obtained from databases detailed in the methods, I would recommend that the authors state clearly the origin of this data in this figure as well or at least refer the potential reader to the methods for the origin of this data.

**Response:**

- Thank you for your kind comments. We have supplemented the description of these data sources in the **Methods** section of the manuscript with references to make it easier for potential readers to refer to these data sources (“*We leveraged the adipose tissue single-cell RNA sequencing data provided by the Tabula Sapiens Consortium to identify the expression levels of the genes in different cell types(5). The Tabula Sapiens is a single-cell transcriptomic atlas of 24 human tissues and organs from 15 unique donors. The adipose tissues from two donors were obtained from surgery and immediately prepared from a fluorescence-activated cell sorting-sorted smart-seq2 platform or 10x Genomics 3' V3.1 droplet-based sequencing platform. The quality control, data integration, clustering, and cell type annotation procedures of single-cell RNA sequencing data were conducted, as described in the previous study(5)*”).
- **Reference**:

5.	Jones RC, Karkanias J, Krasnow MA, Pisco AO, Quake SR, Salzman J, et al. The Tabula Sapiens: A multiple-organ, single-cell transcriptomic atlas of humans. Science (New York, NY). 2022;376(6594):eabl4896.

Minor comments

1) Throughout the manuscript "whole exome sequencing" and "whole-exome sequencing" are used interchangeably, I would recommend the authors pick one form and edit the manuscript accordingly.

**Response:**

- Thank you for your kind comments. We uniformly chose the "*whole-exome sequencing*" format for editing the manuscript.

2) Line 54 Author Summary/Line 82-83 Introduction - The authors state "Family history is one of the important risk factors for VVs, which suggests that VVs are likely modulated by genetic factors." This is an ambiguous statement to make in my opinion, are the authors referring to a family history of VV or a general family history, also if this is one of the important risk factors what are the other risk factors or is this the most important factor? I recommend that the authors amend this statement to make it clearer.

**Response:**

- Thank you for your kind comments. Based on your suggestion, we have decided to delete this ambiguous sentence in the **Author Summary** and change this sentence in the **Introduction** to "*In addition to this, a positive family history is one of the important risk factors for VV, which suggests that VV are likely to be modulated by genetic factors*".

3) In multiple figure/table legends the abbreviation defined include "Lof, loss of function" when this abbreviation has not been used in the associated figure table. Found in Table 1, Figure 1. Please amend.

**Response:**

- Thank you for your kind comments. We have removed the abbreviation "Lof, Loss of Function" from the legends to **Table 1** and **Fig. 1**.

4) line 81 - "associated with some health risk factors, including advanced age, female, pregnancy, obesity, high height, and..." consider change to "associated with several risk factors, including advanced age, being female (or sex of the individual), pregnancy, obesity, height, and..."

**Response:**

- Thank you for your kind comments. We have modified this ambiguous expression in response to your suggestion in **Introduction** and changed it to “*Previous epidemiological studies have shown that VV are associated with several risk factors, including advanced age, being female (or sex of the individual), pregnancy, obesity, height, and history of deep vein thrombosis*”.

5) Line 314 - "results did not change obviously after" I recommend the authors revise the usage of "obviously" in this context and rephrasing this sentence in a for a more objective manner.

**Response:**

- Thank you for your kind comments. We have changed the description of "results did not change obviously after" in the **Discussion** and replaced it with "...*results remained stable after...*".

6) Lines 331-333. The sentence starting "Nevertheless, previous studies using the same..." could be re-ordered for clarity as something like "Nevertheless, previous studies have been able to replicate genetic loci findings for common variants using the phenotypic definitions in GWAS analyses."

**Response:**

- Thank you for your kind comments. We have changed the sentence beginning with "Nevertheless, previous studies using the same..." to "*Nevertheless, previous studies have been able to replicate genetic loci findings for common variants using the phenotypic definitions in GWAS analyses*" in the **Discussion**.

7) The text in the Material and Methods section needs to be revised as there are several sentences not conforming to proper grammatical English and too many sentences to individual point out that are poorly constructed in which essential words are missing.

**Response:**

- Thank you for your kind comments. We have made grammatical modifications and textual changes to the presentation of the **Methods** sections.

**Response to Reviewer #2**

Reviewer #2

In this study, Zhang et al. investigate the genetic architecture of varicose veins (VV) through the analysis of whole-exome sequencing data in the UK Biobank. Overall, the authors describe a substantial number of new associations with several genes, both from common and rare variant analyses. Coupled with gene set enrichment and expression analyses, the authors provide useful biological interpretation of these findings which help in better understand the disease. However, I have some comments on the manuscript before it can be accepted to publication.

Major comments

This study is not the first association study performing rare variant burden analyses. Van Hout et al. have indeed investigated associations between rare variants and multiple phenotypes, including varicose veins, through LoF burden analyses (10.1038/s41586-020-2853-0). The authors should at least compare their results to this study.

**Response:**

- Thank you for your kind comments. As per your suggestion, we have added a comparison of the present study with that of Van et al. in the first paragraph of the **Discussion** section (“*In a recent WES study on the phenotype-wide association(6), a PIEZO1 gene significantly associated with VV was identified by utilizing exome-wide data from nearly 50,000 people from UKB. In comparison to the Van et al. study, our study had a more comprehensive definition of VV, a larger sample size, better subsequent analysis, and not only validated the PIEZO1 it found but additionally identified other genes associated with VV*”).
- **Reference**:

6.	Van Hout CV, Tachmazidou I, Backman JD, Hoffman JD, Liu D, Pandey AK, et al. Exome sequencing and characterization of 49,960 individuals in the UK Biobank. Nature. 2020;586(7831):749-56.

Age being an important risk factor of the disease, it would be useful to have the age distribution in cases and controls in addition of the overall age statistics given in l. 113. If there is a large difference between cases and controls and age is included as a covariate, then it could mask some signals. If this is the case, I would suggest performing a sensitivity analysis with a selection of controls to have a similar age distribution as the cases as the number of controls shouldn’t be an issue in the UK Biobank.

**Response:**

- Thank you for your kind comments. We matched the VV case and control groups on the basis of age, as you suggested, so that the age distributions of the two populations were similar (**Addiotional file 3. Supplementary Fig. S5**, **Methods – Sensitivity and leave-one-variant-out analyses**: “*Furthermore, since age is also one of the important risk factors for the disease, we performed a sensitivity analysis after age-matching the case and control groups according to 1:10*”). We then performed sensitivity analyses on these age-matched populations, which are described in the **Results** section of the manuscript (**Results - Sensitivity analysis of the effects of age and gender on VV**: “*To minimize the effect of age distribution on the results, we matched the case and control groups 1:10 by age (Additional file 3. Fig. S4). In the age-matched population, a total of 168 variants were found to reach full exonic significance in the single-variant analysis (Additional file 12, P < 1.19 × 10^-6^), which was almost indistinguishable from the total population (169 variants). Collapse analyses were also performed in the matched population and showed that PIEZO1 and FBLN7 were still found to be correlated with VV (Additional file 13, P < 2.51 × 10^−6^)*”).

Why is the sex-specific analysis only performed with the rare variants and not with the common ones using single-point associations? It would be interesting to see whether additional variants come up when studying the common variants and whether there is also a signal in METTL21A.

**Response:**

- Thank you for your kind comments. Following your suggestion, we also performed sex-specific analyses of common variants in single-variant associations (**Methods - Sensitivity and leave-one-variant-out analyses**: “*…we performed single-variant association analyses and gene-based collapse analyses on participants of different genders to…*”). The results show that 4 additional novel genes (*ARHGEF26*, *BTN1A1*, *H1-5*, *TRIM27*) were identified in females only, which did not include the *METTL21A* gene found in the rare variants (**Results - Sensitivity analysis of the effects of age and gender on VV**: “*After clumping, we identified 8 and 21 common variants with significant associations with VV in males and females, respectively, with 4 variants mapping to novel genes only in females (ARHGEF26, BTN1A1, H1-5, TRIM27,* ***Additional file 15****)*”).

Some clarifications on the methods used are needed:

For the common single-point associations, the significance threshold was computed based on the Bonferroni correction, but was the correlation between the variants considered to compute this threshold? It would also be interesting to report the number of signals significant at the genome-wide significance threshold. Actually, most of the signals that are not replicated in the FinGen cohort are not significant at the genome-wide level.

**Response:**

- Thank you for your kind comments. The significance thresholds calculated in this paper for common single-point variants according to the Bonferroni correction are based on previous papers(7, 8) that do not mention the consideration of correlation between variants. In addition, as you suggested, we added a solid line indicating the genome-wide significance threshold (*P* = 5 × 10^-8^) in **Fig. 1a** to be able to visualize the signals that are still significant. The results showed that there were 114 variants reached the genome-wide significance.
- **Reference**:

7.	Singh T, Poterba T, Curtis D, Akil H, Al Eissa M, Barchas JD, et al. Rare coding variants in ten genes confer substantial risk for schizophrenia. Nature. 2022;604(7906):509-16.

8.	Chen CY, Tian R, Ge T, Lam M, Sanchez-Andrade G, Singh T, et al. The impact of rare protein coding genetic variation on adult cognitive function. Nat Genet. 2023;55(6):927-38.

What significance threshold was used for the replication cohort?

**Response:**

- Thank you for your kind comments. The significant threshold we used for the replication cohort was *P*-value < 0.05.

Can the authors give more details about the collapsing analyses? Which burden score and which software were used to perform these analyses? In addition, it would be useful in table 2 to have the number of variants retained in each testing unit to compare the results of the different masks.

**Response:**

- Thank you for your kind comments. The software we used to perform collapsing analysis were “SAIGE-GENE+”, which presented in “**Methods: Gene-based exome-wide association analysis:** *ExWAS was conducted in SAIGE-GENE+ software(9) with a logistic mixed model*”. In addition, we have added the number of variants in each mask unit in **Table 2** as per your suggestion.
- **Response**:

9.	Zhou W, Bi W, Zhao Z, Dey KK, Jagadeesh KA, Karczewski KJ, et al. SAIGE-GENE+ improves the efficiency and accuracy of set-based rare variant association tests. Nat Genet. 2022;54(10):1466-9.

l.211: on which set of genes the gene enrichment analysis was performed?

**Response:**

- Thank you for your kind comments. We performed an enrichment analysis of all 36 genes associated with VV identified in the text. And we add a description of this in the **Results** (“*We conducted a pathway enrichment analysis using the Gene Ontology Consortium for all 36 genes identified by the study as being significantly associated with VV*”).

For the Phe-WAS analysis, what exactly is tested? Does it focus on single-variant associations? Also, what is the exact significance threshold? There is a discrepancy between the methods and the figures. I guess there is a typo, at least in Fig. 4 where it is stated that the threshold is corrected for 3 genes while more than 3 genes are analyzed.

**Response:**

- Thank you for your kind comments. Phe-WAS analysis focused on testing whether genes associated with VV are also associated with other phenotypes. The significance thresholds for Phe-WAS were 2.77 × 10^-5^ and 3.14 × 10^-4^ for common and rare variant genes, respectively. We apologized for the misrepresentation of the legend in **Fig. 4** and have corrected it to read "(...*threshold for 53 traits and 34 genes (P = 2.77×10^-5^)*)".

Can the authors explain in a little bit more details how they computed the disease prevalence for each variant? How did they combine LoF and missense variants?

**Response:**

- Thank you for your kind comments. We calculated the percentage of VV cases among carriers of these rare variants as the disease prevalence of the VV susceptibility gene. We categorize variants that appear either as LOF or missense as combining LOF and missense variants.

More details on the Gene-SCOUT analysis would also help the reader.

**Response:**

- Thank you for your kind comments. We provide specific Gene-SCOUT URL links with references in the **Methods** of the manuscript for readers to better understand it (**Methods: Gene-SCOUT**: “*The Gene-SCOUT (Gene-Similarity from Continuous Traits, https://astrazeneca-cgr-publications.github.io/gene-scout/)(10) aims to...*”)).
- **Reference**:

10.	Middleton L, Harper AR, Nag A, Wang Q, Reznichenko A, Vitsios D, et al. Gene-SCOUT: identifying genes with similar continuous trait fingerprints from phenome-wide association analyses. Nucleic Acids Res. 2022;50(8):4289-301.

There are some sentences in the text that I not fully agree with or that would need additional clarification:

l.96: how rare variants can help in evaluating disease prevalence?

**Response:**

- Thank you for your kind comments. What we are trying to convey in the manuscript is that large-scale sequencing can be used to assess disease prevalence and carrier frequencies of rare genetic variants, rather than that rare variants can help assess disease prevalence.

l.88: how rare variants can more directly inform causal genes? At the end, GWAS and collapsing tests are both association tests, the difference being in how the variants are analyzed.

**Response:**

- Thank you for your kind comments. Previous studies(6, 11-13) have shown that rare coding variants (MAF < 0.01) tend to show immediate biological effects, pinpoint the causative gene, and provide guidance for subsequent experiments compared to common variants.
- **Reference**:

6.	Van Hout CV, Tachmazidou I, Backman JD, Hoffman JD, Liu D, Pandey AK, et al. Exome sequencing and characterization of 49,960 individuals in the UK Biobank. Nature. 2020;586(7831):749-56.

11.	Fiziev PP, McRae J, Ulirsch JC, Dron JS, Hamp T, Yang Y, et al. Rare penetrant mutations confer severe risk of common diseases. Science (New York, NY). 2023;380(6648):eabo1131.

12.	Backman JD, Li AH, Marcketta A, Sun D, Mbatchou J, Kessler MD, et al. Exome sequencing and analysis of 454,787 UK Biobank participants. Nature. 2021;599(7886):628-34.

13.	Ruth KS, Day FR, Hussain J, Martínez-Marchal A, Aiken CE, Azad A, et al. Genetic insights into biological mechanisms governing human ovarian ageing. Nature. 2021;596(7872):393-7.

I think there is a confusion between the effect sizes and the p-values. Indeed, it says at l. 151 that effect sizes are enhanced by the inclusion of missense variants. This is not true when looking at table 2 where p-values are indeed lower when missense are included, but the effect sizes (OR) are smaller. Similarly, in lines 204-205, the authors mention the different effect sizes but report only p-values and not OR.

**Response:**

- Thank you for your kind comments. We have re-examined this statement and apologize for the misrepresentation of the effect size and have changed it to "*The significant level of the genetic variation associations between these significant genes and VV was enhanced by the inclusion of missense variants*" in the **Results - Three VV genes identified by collapse analysis** and “*The PIEZO1 gene was strongly associated with VV, with different significant levels across genders (male, P = 4.66 × 10^-14^; female, P = 6.81 × 10^-21^)*” in the **Results - Robustness of rare variant gene-VV associations**.

What do the authors mean by “genetic variants identified in population-based whole exome-sequencing studies were more reliable” (l. 255)? It has been actually showed that rare variant analyses are prone to more biased compared to common variants.

**Response:**

- Thank you for your kind comments. Whole-exome sequencing study (WES) is often the result of data obtained directly through sequencing, rather than as a result of interpolation. Moreover, WES enables direct assessment of variants that alter proteins with functional consequences that are easier to interpret than non-coding variants, providing a clearer pathway to deeper mechanistic and therapeutic insights and potentially for therapeutic target discovery and validation(14-17) as well as precision medicine(18, 19).
- **Reference**:

14.	Abul-Husn NS, Cheng X, Li AH, Xin Y, Schurmann C, Stevis P, et al. A Protein-Truncating HSD17B13 Variant and Protection from Chronic Liver Disease. The New England journal of medicine. 2018;378(12):1096-106.

15.	Dewey FE, Gusarova V, Dunbar RL, O'Dushlaine C, Schurmann C, Gottesman O, et al. Genetic and Pharmacologic Inactivation of ANGPTL3 and Cardiovascular Disease. The New England journal of medicine. 2017;377(3):211-21.

16.	Cohen JC, Boerwinkle E, Mosley TH, Jr., Hobbs HH. Sequence variations in PCSK9, low LDL, and protection against coronary heart disease. The New England journal of medicine. 2006;354(12):1264-72.

17.	Scott RA, Freitag DF, Li L, Chu AY, Surendran P, Young R, et al. A genomic approach to therapeutic target validation identifies a glucose-lowering GLP1R variant protective for coronary heart disease. Sci Transl Med. 2016;8(341):341ra76.

18.	Abul-Husn NS, Manickam K, Jones LK, Wright EA, Hartzel DN, Gonzaga-Jauregui C, et al. Genetic identification of familial hypercholesterolemia within a single U.S. health care system. Science (New York, NY). 2016;354(6319).

19.	Manickam K, Buchanan AH, Schwartz MLB, Hallquist MLG, Williams JL, Rahm AK, et al. Exome Sequencing-Based Screening for BRCA1/2 Expected Pathogenic Variants Among Adult Biobank Participants. JAMA network open. 2018;1(5):e182140.

l.319: I wouldn’t say that the overlapping genes are more accurate, it actually depends on the genetic architecture of the trait. It is possible that some genes only present common variation that is associated to the disease.

**Response:**

- Thank you for your kind comments. Following your suggestion, we have replaced the statement in the **Discussion** with "*Moreover, the current study revealed an overlap between the VV-related genes identified by single-variant analyses and those identified by burden tests, suggesting the robustness of the association between these overlapping genes and VV*".

Overall, I would suggest the authors to carefully proofread the manuscript and improve the writing to make things clearer.

**Response:**

- Thank you for your kind comments. We have carefully proofread the manuscript and improved the writing based on your suggestions.

Minor comments:

The single common variant analysis seems a little bit inflated. What is the lambda value?

**Response:**

- Thank you for your kind comments. We calculated the results of the individual common variant analyses based on your suggestion and came up with a lambda value of 0.847.

Why only four masks out of the 8 are reported in table 2?

**Response:**

- Thank you for your kind comments. Based on your suggestions, we have added other four mask results in **Table 2**.

L.134-135: I don’t agree with the sentence that this is especially true for novel signals, we don’t see a large difference in Fig.1 in effect sizes and frequency distributions between novel and previous variants.

**Response:**

- Thank you for your kind comments. We deleted the phrase "especially the 16 novel variants" from the **Results** section of the manuscript and changed the sentence to "*It was noteworthy that these significant variant associations were characterized by larger effect sizes at lower frequencies*". We removed the distinction between the new variants and the previous variants from **Fig. 1c** and showed the effect sizes and frequency distributions for all variants directly.

Would the genes still be significant in collapsing analyses if the significance threshold is corrected by the number of masks used?

**Response:**

- Thank you for your kind comments. Because the low power inherent in type 1 errors is necessarily exacerbated when they are adjusted to control for multiple tests of all 8 crash models, we used Bonferroni corrections only for the total number of genes and made the significance threshold comparable to recent exome sequencing studies(20-22).
- **Reference**:

20.	Choi SH, Jurgens SJ, Weng LC, Pirruccello JP, Roselli C, Chaffin M, et al. Monogenic and Polygenic Contributions to Atrial Fibrillation Risk: Results From a National Biobank. Circ Res. 2020;126(2):200-9.

21.	Huang Y, Bodnar D, Chen CY, Sanchez-Andrade G, Sanderson M, Shi J, et al. Rare genetic variants impact muscle strength. Nature communications. 2023;14(1):3449.

22.	Gao XR, Chiariglione M, Arch AJ. Whole-exome sequencing study identifies rare variants and genes associated with intraocular pressure and glaucoma. Nature communications. 2022;13(1):7376.

In Fig. 3, we see a very distinct signature for some genes such as SEPRRIND1 and SLC17A2 in the liver for example. Is this something known for this gene or something that could be linked to the disease?

**Response:**

- Thank you for your kind comments. We showed in Fig. 1a that both genes are genes associated with VV, with *SEPRRIND1* being a newly identified VV-associated gene and *SLC17A2* being a gene that has been reported in previous studies to be possibly associated with VV. Fig. 3c was designed to demonstrate the tissue-specific expression of these VV-related genes.

l.231: what are the numbers in parentheses?

**Response:**

- Thank you for your kind comments. The numbers in parentheses represent the number of studied phenotypes and traits associated with each gene.

Why is the past tense used in multiple sentences? (l. 283, l.295. l.303 for example)

**Response:**

- Thank you for your kind comments. We changed the tense expressions of these sentences you mentioned and changed these tenses uniformly to the general present tense.

l.404: could the authors show the PCA in a supplementary figure with cases and controls labelled?

**Response:**

- Thank you for your kind comments. We have added presentation plots of PC1 and PC2 labeled cases and controls in the **Additional file 3: Supplementary Information** and illustrated them in the **Results** (“*The clustering of the VV case and control groups in the principal component analysis was shown in Supplementary Fig. 1 (****Additional file 3****)*”).

There are some typos in the text. For example: Keywords: casual genes; l.331: “used”; l. 446: MAF of each variant, not of each gene; l.299: “to treat”

**Response:**

- Thank you for your kind comments. We have corrected these misspellings and rechecked the entire text.

**Response to Reviewer #3**

Reviewer #3:

The authors carried out an association analysis of whole exome sequencing (WES) data of 350,770 unrelated Caucasian UK Biobank participants and phenotype data on varicose veins (21,643 cases and 329,127 controls). Previous studies (e.g. Fukaya 2018, Shadrina 2019, Ahmed 2022 and Levin 2022) used only genotype data which is less suited to assess rare variants. This current study seems to be the first large study based on WES, however, genetic studies targeting the whole exome level are maybe not as unusual as the authors claim.

The authors analysed common and rare variants using single-variant and gene-based analyses. They identified 34 genes by mapping single-variant associations to genes, and 3 genes by carrying out gene-based tests.

The authors identified 19 known and 17 new genes of which most were validated in FinnGen. Maybe there could be more details given how the validation was carried out.

**Response:**

- Thank you for your kind comments. We added a description of FinnGen validation in the **Methods: Replication of genes in the FinnGen cohort** (“*We found GWAS data for VV in FinnGen and then screened it with a threshold of P < 0.05. We then looked for the presence of the same genes as the significantly associated genes for VV found in our study among the VV genes in the FinnGen cohort at P < 0.05 as a validation*”). We also refined the formulation of FinnGen validation in the **Results** (“*The association between 32 genes and VV was successfully validated in FinnGen*”, “*The association of the three genes we identified in the collapse analysis with VV was further successfully validated in the VV genomic data from FinnGen*”). In **Tables 1 and 2** we show the *P*-value of each VV-associated gene in FinnGen.

The authors also carried out additional analyses of burden heritability, robustness of the gene-based gene enrichment and Phe-WAS analyses. The LOVO analysis showed that the association with the PIEZO1 gene seemed to be influenced by the combination of several rare variants, while the other two significant genes were rather influenced by single rare variants.

The main distinction of this study to previous studies is their use of rare variants. The LOVO analysis is a good start, but could there have been additional ways to analysis these variants? Rare variants usually have larger effect sizes, are less likely to be in linkage disequilibrium with other variants, and could be of greater clinical relevance.

**Response:**

- Thank you for your kind comments. Based on previous literatures(6, 8), we found that the common analysis method for rare variants was LOVO analysis, and that rare variants had fewer carriers and small outcome effects for rare unit point variants, and the focus should be on the gene level.
- **Reference**:

6.	Van Hout CV, Tachmazidou I, Backman JD, Hoffman JD, Liu D, Pandey AK, et al. Exome sequencing and characterization of 49,960 individuals in the UK Biobank. Nature. 2020;586(7831):749-56.

8.	Chen CY, Tian R, Ge T, Lam M, Sanchez-Andrade G, Singh T, et al. The impact of rare protein coding genetic variation on adult cognitive function. Nat Genet. 2023;55(6):927-38.

Maybe variant annotation such as LoF or missense could be added to Table 1. Is there anything more known about particular effects of the LoF and missense variants? It would be interesting to understand what effect the missense variants have on the protein. Additionally, it was not quite clear which cut-offs were used for the missense annotation.

**Response:**

- Thank you for your kind comments. Following your suggestion, we have added some detailed information about important common variants in **Table 1**. In addition, the common variants were annotated by ANNOVAR, and we have added the exonic function of each variant in **Table 1**.

I am not sure whether Figure 3 supports the claim that the PIEZO1 and ECE1 genes were expressed in adipose tissue at significantly higher levels than in other tissues. Figure 3b and 3c seem somewhat

non-specific in terms of tissue enrichment. Additionally, the legend of Figure 3a does not explain the listed categories.

**Response:**

- Thank you for your kind comments. The expression in our manuscript was not rigorous enough, and we replaced the expression in the **Results: Enrichment analysis of VV genes and their expression** with "*PIEZO1 and the newly identified ECE1 gene were also expressed in adipose tissue at relatively higher levels*". Fig. 3b was to show the overall expression level of all VV-associated genes, while Fig. 3c showed the tissue expression level of each gene separately. We supplemented the categorization of Fig. 3a in the legend with Fig. 3 (“*Abbreviation: BP, biological process; CC, cellular component; MF, molecular function*”).

Gene-SCOUT was used to identify two genes, SCUBE3 and IGF2BP2, that were negatively correlated to height-related traits similar to PIEZO1. Was it also used to investigate the other significant genes?

**Response:**

- Thank you for your kind comments. We have tried to find genes similar to *ECE1* and *FBLN7* through gene scouting, but due to the limitations of Gene-SCOUT, no results related to these two genes were found.

Overall, the text would benefit from some grammar checks and re-phrasing, and a more structured and more focused discussion would be easier to read.

**Response:**

- Thank you for your kind comments. We have carefully proofread the manuscript and improved the writing based on your suggestions.

Minor comments:

-------------------

There are some typos in the main text and the legend of Figure 4. Some references and brief explanations in the main text were missing, e.g. for FUMA and DAVID. Additionally, a reference is missing for the claim that a Mendelian randomization study found a positive correlation between adiposity and the risk of VVs.

**Response:**

- Thank you for your kind comments. We have added the full names of FUMA and DAVID with references in the **Methods: Gene set enrichment and expression analyses** (“*the Database for Annotation, Visualization, and Integrated Discovery (DAVID)(23)*”; “*...in Functional Mapping and Annotation of Genome-Wide Association Studies (FUMA GWAS)(24)*”). We have changed the discussion from "A Mendelian randomization study found a positive correlation..." to "*Several studies found a positive correlation between adiposity and the risk of VV(2, 25)*" and added references.
- **Reference**:

2.	Fukaya E, Flores AM, Lindholm D, Gustafsson S, Zanetti D, Ingelsson E, et al. Clinical and Genetic Determinants of Varicose Veins. Circulation. 2018;138(25):2869-80.

23.	Sherman BT, Hao M, Qiu J, Jiao X, Baseler MW, Lane HC, et al. DAVID: a web server for functional enrichment analysis and functional annotation of gene lists (2021 update). Nucleic Acids Res. 2022;50(W1):W216-w21.

24.	Watanabe K, Taskesen E, van Bochoven A, Posthuma D. Functional mapping and annotation of genetic associations with FUMA. Nature communications. 2017;8(1):1826.

25.	Shadrina AS, Sharapov SZ, Shashkova TI, Tsepilov YA. Varicose veins of lower extremities: Insights from the first large-scale genetic study. PLoS genetics. 2019;15(4):e1008110.

Weiner DJ et al. (Nature 2023) appears twice in the references and it was referenced with a typo in main text 'Winer'.

**Response:**

- Thank you for your kind comments. We re-examined the citation of references, removed duplicates from the **References**, and corrected “*Weiner DJ et al*” in the **Results**.

**References:**

1.	Zhou GJ, Zhang Y, Wang J, Guo JH, Ni J, Zhong ZM, et al. Cloning and characterization of a novel human RNA binding protein gene PNO1. DNA sequence : the journal of DNA sequencing and mapping. 2004;15(3):219-24.

2.	Fukaya E, Flores AM, Lindholm D, Gustafsson S, Zanetti D, Ingelsson E, et al. Clinical and Genetic Determinants of Varicose Veins. Circulation. 2018;138(25):2869-80.

3.	Graef IA, Chen F, Chen L, Kuo A, Crabtree GR. Signals transduced by Ca(2+)/calcineurin and NFATc3/c4 pattern the developing vasculature. Cell. 2001;105(7):863-75.

4.	Lek M, Karczewski KJ, Minikel EV, Samocha KE, Banks E, Fennell T, et al. Analysis of protein-coding genetic variation in 60,706 humans. Nature. 2016;536(7616):285-91.

5.	Jones RC, Karkanias J, Krasnow MA, Pisco AO, Quake SR, Salzman J, et al. The Tabula Sapiens: A multiple-organ, single-cell transcriptomic atlas of humans. Science (New York, NY). 2022;376(6594):eabl4896.

6.	Van Hout CV, Tachmazidou I, Backman JD, Hoffman JD, Liu D, Pandey AK, et al. Exome sequencing and characterization of 49,960 individuals in the UK Biobank. Nature. 2020;586(7831):749-56.

7.	Singh T, Poterba T, Curtis D, Akil H, Al Eissa M, Barchas JD, et al. Rare coding variants in ten genes confer substantial risk for schizophrenia. Nature. 2022;604(7906):509-16.

8.	Chen CY, Tian R, Ge T, Lam M, Sanchez-Andrade G, Singh T, et al. The impact of rare protein coding genetic variation on adult cognitive function. Nat Genet. 2023;55(6):927-38.

9.	Zhou W, Bi W, Zhao Z, Dey KK, Jagadeesh KA, Karczewski KJ, et al. SAIGE-GENE+ improves the efficiency and accuracy of set-based rare variant association tests. Nat Genet. 2022;54(10):1466-9.

10.	Middleton L, Harper AR, Nag A, Wang Q, Reznichenko A, Vitsios D, et al. Gene-SCOUT: identifying genes with similar continuous trait fingerprints from phenome-wide association analyses. Nucleic Acids Res. 2022;50(8):4289-301.

11.	Fiziev PP, McRae J, Ulirsch JC, Dron JS, Hamp T, Yang Y, et al. Rare penetrant mutations confer severe risk of common diseases. Science (New York, NY). 2023;380(6648):eabo1131.

12.	Backman JD, Li AH, Marcketta A, Sun D, Mbatchou J, Kessler MD, et al. Exome sequencing and analysis of 454,787 UK Biobank participants. Nature. 2021;599(7886):628-34.

13.	Ruth KS, Day FR, Hussain J, Martínez-Marchal A, Aiken CE, Azad A, et al. Genetic insights into biological mechanisms governing human ovarian ageing. Nature. 2021;596(7872):393-7.

14.	Abul-Husn NS, Cheng X, Li AH, Xin Y, Schurmann C, Stevis P, et al. A Protein-Truncating HSD17B13 Variant and Protection from Chronic Liver Disease. The New England journal of medicine. 2018;378(12):1096-106.

15.	Dewey FE, Gusarova V, Dunbar RL, O'Dushlaine C, Schurmann C, Gottesman O, et al. Genetic and Pharmacologic Inactivation of ANGPTL3 and Cardiovascular Disease. The New England journal of medicine. 2017;377(3):211-21.

16.	Cohen JC, Boerwinkle E, Mosley TH, Jr., Hobbs HH. Sequence variations in PCSK9, low LDL, and protection against coronary heart disease. The New England journal of medicine. 2006;354(12):1264-72.

17.	Scott RA, Freitag DF, Li L, Chu AY, Surendran P, Young R, et al. A genomic approach to therapeutic target validation identifies a glucose-lowering GLP1R variant protective for coronary heart disease. Sci Transl Med. 2016;8(341):341ra76.

18.	Abul-Husn NS, Manickam K, Jones LK, Wright EA, Hartzel DN, Gonzaga-Jauregui C, et al. Genetic identification of familial hypercholesterolemia within a single U.S. health care system. Science (New York, NY). 2016;354(6319).

19.	Manickam K, Buchanan AH, Schwartz MLB, Hallquist MLG, Williams JL, Rahm AK, et al. Exome Sequencing-Based Screening for BRCA1/2 Expected Pathogenic Variants Among Adult Biobank Participants. JAMA network open. 2018;1(5):e182140.

20.	Choi SH, Jurgens SJ, Weng LC, Pirruccello JP, Roselli C, Chaffin M, et al. Monogenic and Polygenic Contributions to Atrial Fibrillation Risk: Results From a National Biobank. Circ Res. 2020;126(2):200-9.

21.	Huang Y, Bodnar D, Chen CY, Sanchez-Andrade G, Sanderson M, Shi J, et al. Rare genetic variants impact muscle strength. Nature communications. 2023;14(1):3449.

22.	Gao XR, Chiariglione M, Arch AJ. Whole-exome sequencing study identifies rare variants and genes associated with intraocular pressure and glaucoma. Nature communications. 2022;13(1):7376.

23.	Sherman BT, Hao M, Qiu J, Jiao X, Baseler MW, Lane HC, et al. DAVID: a web server for functional enrichment analysis and functional annotation of gene lists (2021 update). Nucleic Acids Res. 2022;50(W1):W216-w21.

24.	Watanabe K, Taskesen E, van Bochoven A, Posthuma D. Functional mapping and annotation of genetic associations with FUMA. Nature communications. 2017;8(1):1826.

25.	Shadrina AS, Sharapov SZ, Shashkova TI, Tsepilov YA. Varicose veins of lower extremities: Insights from the first large-scale genetic study. PLoS genetics. 2019;15(4):e1008110.
