# Supplementary material for: Exome sequencing identifies novel genetic variants associated with varicose veins
Source: PLoS Genet. 2024 Jul 9;20(7):e1011339. doi: 10.1371/journal.pgen.1011339 (PMC11233024; doi:10.1371/journal.pgen.1011339)
Supplement: Attachment — Submitted filename: Review_R1_PlosG_2024.docx [file pgen.1011339.s029.docx]

I thank the authors for considering the comments made on their manuscript and for adding analyses in the new version. However, I find that not of them have been addressed (example of minor comments 4 and 5), and when they have, the text was not necessarily modified accordingly. In my opinion, the manuscript is still unclear in several places, especially where clarification was asked in the comments. I have an additional set of comments for the authors to address:

**Major comments**

1. While the authors have mentioned the previous study by Van Hout et al., it has only been done in the discussion and not the introduction. This study should be mentioned in the introduction to clearly make the state of the art, as for now it seems that this study is the first collapsing study on VV, which is not true. In addition, the study from Van Hout et al. is based on the same UKBB cohort, and the one from Zhang et al. represent an extension of it. I therefore think that a more comprehensive comparison of effect sizes or p-values across the two studies should be done rather than a simple sentence in the discussion. Similarly, l.363, I do not agree that ‘WES of VV was lacking’.
2. The PCA in Fig. S1 is useful to see that cases and controls overlap and that there is probably no bias in the analysis but why the legend of the figure indicates epilepsy group vs non-epilepsy group?! In addition, why -log10 of the PCs are represented and not simply the PC values?
3. In table 2, how the number of rare variants can be lower than the number of ultra-rare variants? What is the definition for these two categories? I find the number of ultra-rare variants very high given the masks applied.
4. In response to the authors: “Whole-exome sequencing study (WES) is often the result of data obtained directly through sequencing, rather than as a result of interpolation. Moreover, WES enables direct assessment of variants that alter proteins with functional consequences that are easier to interpret than non-coding variants, providing a clearer pathway to deeper mechanistic and therapeutic insights and potentially for therapeutic target discovery and validation(14-17) as well as precision medicine(18, 19).”

- I agree with the authors, but this is still not clear in the text as nothing has been modified. There are two things here: (1) accessing rare variation through sequencing rather than imputation, which indeed leads to a higher confidence in the genotypes; and (2) accessing coding versus non-coding variation, so WES vs WGS. In the text, it is stated ‘more reliable’ but with no mention to which this is compared.

**Minor comments**

- The number of signals significant at the genome-wide threshold can be indicated in the text in addition of the red line on the Manhattan plot.
- It would be useful to have the lambda values on the QQ-plot in addition of only a response to this question in the response. Also, I find that the points are very large on the QQ-plots, I would decrease their size to have a better visualization.
- I thank the authors for adding the age-matched analysis. However, given that the age distribution between the cases and controls was already quite similar, I don’t think this analysis was needed, the authors could have provided only the left graph of Fig. S5.
- I would refer to ‘significance level’ rather than ‘significant level’.
- L.134: this section is still about the single-point associations and not the collapsing tests. It would be better to report the number of replicated variants rather than the number of replicated genes, this is confusing with the collapsing tests.
- In table 2, does “/” represent the variants not replicated or not tested in FinGen? If the former, why isn’t the p-value of the test provided?
- L. 282: what does ‘better subsequent analysis’ mean?
- L. 455: I think the authors refer to ‘SAIGE’ and not ‘SAIGE-GENE+’ for single point associations.
- Out of curiosity, can the authors explain why they used the mixed models and not the saddle-point approximation as there is a case-control unbalance and as relatedness has been considered in QC?
- L. 214: simply as a suggestion, I think that a conditional analysis like COJO instead of a clumping would be useful.
- L.220-225: I still think that biological insight is missing from the described sex-specific associations and how it can help in better understanding the etiology of VV.
