# Supplementary material for: Exome sequencing identifies novel genetic variants associated with varicose veins
Source: PLoS Genet. 2024 Jul 9;20(7):e1011339. doi: 10.1371/journal.pgen.1011339 (PMC11233024; doi:10.1371/journal.pgen.1011339)
Supplement: Attachment — Submitted filename: Response to Reviewers.docx [file pgen.1011339.s030.docx]

The authors sincerely appreciate the critical reviews of the paper, and for the helpful way in which the reviewing editors put together a constructive list of suggestions for the revision of the paper. We have now revised the paper to carefully address all the points raised. Our responses below are preceded by “-”, and changes made to the paper are shown below within “…”, and in red font in the revised paper.

**Detailed Response to Editor**

- Many thanks for your positive feedback and for your high concern towards our manuscript. The reviewers' valuable comments indeed provided important guidance to our research. By thoughtfully integrating their insights, we revised the paper and believe the manuscript's quality has been improved.

**Detailed Response to Reviewers**

**Response to Reviewer #1**

The authors of the manuscript have taken the suggestions and comments from all reviewers to their manuscript. Having for the majority answer them successfully, the authors have now improved their manuscript which following some more minor revisions should be ready for publication.

Minor revision points to consider:

Line 80 - In answering my previous Minor comment 4) the authors have included both my suggestions when my intent was for the authors to pick one (either being female or the sex of individual). I apologize for not being clearer in my original comment but recommend the authors make a minor edit to this sentence one final time.

**Response:**

- Thank you for your kind comments. We have modified this ambiguous expression in response to your suggestion in **Introduction** and changed it to “*Previous epidemiological studies have shown that VV are associated with several risk factors, including advanced age, being female, pregnancy, obesity, height, and history of deep vein thrombosis*”.

Line 282 - The portion of the sentence "and not only validated the PIEZO1 it found but additionally identified other genes associated with VV." needs some minor editing to change it into correct grammatical sense.

**Response:**

- Thank you for your kind comments. We have changed this expression in **Discussion** and changed it to “*Compared to the studies by Van Hout et al. and Wang et al., our study has a larger sample size and more validation of the findings (replication in the FinnGen cohort) and exploration of biological function (e.g. functional enrichment analysis, tissue expression analysis, and single-cell RNA sequencing analysis)”*.

Line 308 - The authors used the expression "3 rare genes" in this sentence. I question whether the genes are rare or if the variants identified in these genes are rare? Please amend.

**Response:**

- Thank you for your kind comments. We revised this ambiguous expression in **Discussion** and changed it to “*The other findings were the associations of VV with three rare variant genes (PIEZO1, ECE1, and FBLN7) identified by the gene-based collapsing analysis*”.

**Response to Reviewer #2**

Reviewer #2

I thank the authors for considering the comments made on their manuscript and for adding analyses in the new version. However, I find that not of them have been addressed (example of minor comments 4 and 5), and when they have, the text was not necessarily modified accordingly. In my opinion, the manuscript is still unclear in several places, especially where clarification was asked in the comments. I have an additional set of comments for the authors to address:

Major comments

1) While the authors have mentioned the previous study by Van Hout et al., it has only been done in the discussion and not the introduction. This study should be mentioned in the introduction to clearly make the state of the art, as for now it seems that this study is the first collapsing study on VV, which is not true. In addition, the study from Van Hout et al. is based on the same UKB cohort, and the one from Zhang et al. represent an extension of it. I therefore think that a more comprehensive comparison of effect sizes or p-values across the two studies should be done rather than a simple sentence in the discussion. Similarly, l.363, I do not agree that ‘WES of VV was lacking’.

**Response:**

Thank you for your kind comments. We have added a description of the Van Hout et al. study and outlined the differences from our study in **Introduction** (“*A multi-phenotype exome-wide association study (ExWAS) from the UK Biobank (UKB) involving 49,960 participants identified novel LOF variants with significant disease impact, including PIEZO1 on VV (P=3.2 × 10^-8^). In addition, a phenotype-wide level ExWAS exploring the contribution of rare variants to human disease also found the association between PIEZO1 and VV (P = 3.24 × 10^-24^). In contrast to their studies, we conducted a more comprehensive ExWAS of VV using the updated exome sequencing data of more than 350,000 UKB individuals, with additional validation and exploration of the findings and biological functions*”) and **Discussion** (“*Recently, several studies of human gene-phenotype association for rare coding variants have identified the association between PIEZO1 and VV by using exome sequencing data from UKB participants.* *Compared to the studies by Van Hout et al. and Wang et al., our study has a larger sample size and more validation of the findings (replication in the FinnGen cohort) and exploration of biological function (e.g. functional enrichment analysis, tissue expression analysis, and single-cell RNA sequencing analysis)*”). Furthermore, we apologize for the misunderstanding caused by our misrepresentation, and we have changed “WES of VV was lacking” to "*Due to the difficulty in obtaining exome sequencing data for VV from other databases, we validated our findings using summary statistics from FinnGen*" in **Discussion**.

2) The PCA in Fig. S1 is useful to see that cases and controls overlap and that there is probably no bias in the analysis but why the legend of the figure indicates epilepsy group vs non-epilepsy group?! In addition, why -log10 of the PCs are represented and not simply the PC values?

**Response:**

- Thank you for your kind comments. We apologize for misrepresenting the legend and have changed **Fig. S1** to **Fig. S2**. We have changed the wrong legend to "*Principal component plots of the VV group versus the non-VV group*" in **Fig. S2** and redrawn the graphs using the first and second PC values for presentation. Please see **Fig. S2** as below.

3) In table 2, how the number of rare variants can be lower than the number of ultra-rare variants? What is the definition for these two categories? I find the number of ultra-rare variants very high given the masks applied.

**Response:**

- Thank you for your kind comments. We apologize for causing misunderstanding among reviewers. The definitions of rare variant and ultra-rare variant in our study are not the same as those in SAIGE-GENE+ (<https://saigegit.github.io/SAIGE-doc/docs/set_step2.html>). Rare variants in our study are defined as those with MAF < 0.01, and the number of rare variants in SAIGE-GENE+ and **Table 2** refers to the number of markers that are not ultra-rare with minor allele count in the set (MAC) > 10. The data presented in **Table 2** are the original result in SAIGE-GENE+, where ultra-rare refers to the default setting and is defined as MAC ≤ 10. We have apologetically generalized the number of rare variants in **Table 2** to apply to the definition of rare variants in our study and placed the definition of rare variants at the bottom of **Table 2** (“*Number of rare variants: number of markers that MAF < 0.01*”).

4) In response to the authors: “Whole-exome sequencing study (WES) is often the result of data obtained directly through sequencing, rather than as a result of interpolation. Moreover, WES enables direct assessment of variants that alter proteins with functional consequences that are easier to interpret than non-coding variants, providing a clearer pathway to deeper mechanistic and therapeutic insights and potentially for therapeutic target discovery and validation(14-17) as well as precision medicine(18, 19).”

I agree with the authors, but this is still not clear in the text as nothing has been modified. There are two things here: (1) accessing rare variation through sequencing rather than imputation, which indeed leads to a higher confidence in the genotypes; and (2) accessing coding versus non-coding variation, so WES vs WGS. In the text, it is stated ‘more reliable’ but with no mention to which this is compared.

**Response:**

- Thank you for your kind comments. Based on your suggestion we have added an explanation in **Discussion** (“*VV-related genes and genetic variants identified in population-based WES studies were more abundant and reliable. It is perhaps that whole-exome data are usually obtained directly from sequencing rather than by imputation. WES typically focuses on coding regions of the genome and is more cost-effective and widely available than whole-genome sequencing. Compared to non-coding variants, WES can directly assess variants that alter protein sequence, making it easier to explain their functional consequences. It also provides a clearer pathway to deeper mechanistic insights, and can potentially be help for therapeutic target discovery and precision medicine*”).

Minor comments

The number of signals significant at the genome-wide threshold can be indicated in the text in addition of the red line on the Manhattan plot.

**Response:**

- Thank you for your kind comments. Following your suggestion, we have indicated the number of signals significant at genome-wide thresholds in **Results**: “*Firstly, 169 variants and 114 variants were found to reach the exome-wide significance (P < 1.19 × 10^-6^) and the genome-wide significance (P < 5 × 10^-8^) in the single-variant analysis (****Fig. 1a, Additional file 4****)*”.

It would be useful to have the lambda values on the QQ-plot in addition of only a response to this question in the response. Also, I find that the points are very large on the QQ-plots, I would decrease their size to have a better visualization.

**Response:**

- Thank you for your kind comments. Based on your suggestion, we have added the lambda values on the QQ-plot and decreased the size of the points on the QQ-plot to get a better visualization. The Q-Q pltos (**Fig. 1b** (left) and **Fig. S3** (right)) are shown as below.

I thank the authors for adding the age-matched analysis. However, given that the age distribution between the cases and controls was already quite similar, I don’t think this analysis was needed, the authors could have provided only the left graph of Fig. S5.

**Response:**

- Thank you for your kind comments. Following your suggestion, we have removed the age-matched analyses and retained only the left graph of **Fig. S5** to show the age distribution of the case and control groups (changed **Fig. S5** to **Fig. S1**. **Results**: “*The case and control groups had similar age distribution (****Additional file 3. Fig. S1****)*”). **Fig. S1** is shown as below.

I would refer to ‘significance level’ rather than ‘significant level’.

**Response:**

- Thank you for your kind comments. We have changed "significant level" to "*significance level*" in the **Manuscript**.

L.134: this section is still about the single-point associations and not the collapsing tests. It would be better to report the number of replicated variants rather than the number of replicated genes, this is confusing with the collapsing tests.

**Response:**

- Thank you for your kind comments. According to your suggestions, we have changed the expression “The association between 32 genes and VV was successfully validated in FinnGen” to “*After clumping 169 variants in strong linkage disequilibrium, 36 independent common variants associated with VV were identified (****Fig. 1a****), of which 34 were successfully validated in FinnGen (****Table 1, Additional file 5****)*” in **Results**.

In table 2, does “/” represent the variants not replicated or not tested in FinGen? If the former, why isn’t the p-value of the test provided?

**Response:**

- Thank you for your kind comments. The "/" in our table represent the variants not tested in FinnGen, and we have added the explanation at the bottom of the table (“*The “/” represents the variants not tested in FinnGen*”).

L.282: what does ‘better subsequent analysis’ mean?

**Response:**

- Thank you for your kind comments. We have removed the expression of "better follow-up analyses" and changed it to “*Compared to the studies by Van Hout et al. and Wang et al., our study has a larger sample size and more validation of the findings (replication in the FinnGen cohort) and exploration of biological function (e.g. functional enrichment analysis, tissue expression analysis, and single-cell RNA sequencing analysis)*” in **Discussion**.

L.455: I think the authors refer to ‘SAIGE’ and not ‘SAIGE-GENE+’ for single point associations.

**Response:**

- Thank you for your kind comments. We have changed “SAIGE-GENE+” to “*SAIGE*” in **Methods - Single-variant association analysis** at your suggestion.

Out of curiosity, can the authors explain why they used the mixed models and not the saddle-point approximation as there is a case-control unbalance and as relatedness has been considered in QC?

**Response:**

- Thank you for your kind comments. We apologize for the incomplete description in **Methods**. The saddle-point approximation is used in the mixed model approach in SAIGE-GENE+ software and does not require us to set this up additionally. We have provided detailed explanations in **Methods**: “*Following adjustment for age, sex, and the top 10 PCs, gene-based collapsing analysis was conducted in SAIGE-GENE+ software using a logistic mixed model approach with a saddle-point approximation. This approach is effective for the analysis of large sample data, and provides accurate P-values even when the case-control ratio is highly unbalanced*”.

L.214: simply as a suggestion, I think that a conditional analysis like COJO instead of a clumping would be useful.

**Response:**

- Thank you for your kind comments. Following your suggestion, we have added COJO analysis to our study (**Methods - Conditional analysis**: “*We conducted multi-SNP-based conditional and joint association analyses (GCTA-COJO) using GCTA software to identify conditionally independent variants from common variants associated with VV and to validate the results after clumping. The significance threshold was set at P = 1.19 × 10^−6^, and other parameters were left at their default settings*”; **Results – Exome-wide single-variant association analysis for VV:** “*Furthermore, we conducted a conditional analysis of the identified common variants linked to VV using conditional and joint analysis (COJO). The results revealed that 27 independent common variants were retained, 25 of which were consistent with variants identified after clumping (****Additional file 6****)*”).

L.220-225: I still think that biological insight is missing from the described sex-specific associations and how it can help in better understanding the etiology of VV.

**Response:**

- Thank you for your kind comments. Following your suggestion, we have added enrichment analysis of VV-related common variant genes found in different sexes in **Methods** (“*Enrichment analysis was then performed separately for the VV-related genes identified by sex*”) and **Results - Enrichment analysis of VV genes and their expression** (“*In females, GO enrichment analysis showed that the identified VV-related genes were significantly enriched in protein ubiquitination (P = 1.47 × 10^-3^) and most genes were enriched in protein binding (P = 1.48 × 10^-2^) (****Fig. 3b, Additional file 17****). However, no enrichment results were found due to fewer identified VV genes in males*”). **Fig. 3b** is shown as below.

**Response to Reviewer #3**

Reviewer #3

The revised manuscript is an improved version compared to the original submission, however there are still many cases of wrong use of tense and ill-phrased terms and sentences.

- Past tense instead of present tense is being used in some sentences added in the revision, e.g.

L114 - The clustering of the VV case and control groups in the 115 principal component analysis was shown in Fig. S1.

L135 - The quantile-quantile (Q-Q) plots of single common 136 variants were shown in Fig. 1b.

L152 - The corresponding Q-Q plots were shown in Additional file 3. Fig.S2.

L383 - The UKB was a large-scale prospective cohort...

**Response:**

- Thank you for your kind comments. Following your suggestion, we have changed the past tense expression in **Results** and **Methods** to the present tense: “*The clustering of the VV case and control groups in the principal component analysis is shown in* ***Fig. S2*** *(****Additional file 3****)*”, “*The quantile-quantile (Q-Q) plot of single common variants is shown in* ***Fig. 1b***”, “*The corresponding Q-Q plots are shown in* ***Additional file 3.*** ***Fig. S3***”, “*The UKB is a large-scale prospective cohort...*”.

Maybe 'offers' instead of 'offered'.

L312 - A study by Backman et al. found that missense variants in PIEZO1 might have a gain-of-function effect and reduce VV risk(34), which offered the possibility of treating VV by pharmacological interventions.

**Response:**

- Thank you for your kind comments. We have changed the expression “...which offered the possibility...” in **Discussion** to “...*which offers the possibility...*”.

- Probably better described in plural than singular.

L134 - The associations between 32 genes and VV were successfully validated in FinnGen.

L165 - The associations of the three genes we identified in the collapse analysis with VV were further successfully validated in the VV genomic data from FinnGen

**Response:**

- Thank you for your kind comments. Following your suggestion, we have changed the singular expression in **Results** to plural: “*…of which 34 were successfully validated in FinnGen (****Table 1, Additional file 5****)*” and “*The associations of the three genes we identified in collapsing analysis with VV were further successfully validated in the VV genomic data from FinnGen*”.

L141 - 'Not replicated in FinnGen' rather than 'Not duplicated in FinnGen'

**Response:**

- Thank you for your kind comments. Based on your comments and those of other reviewers, we have changed “Not duplicated in FinnGen” to “*The “/” represents the variants not tested in FinnGen*” at the bottom of **Table 1**.

The term 'collapse analysis' sounds slightly odd, especially in the header here.

L 146 - Three VV genes identified by collapse analysis

Maybe better 'rare-variant collapsing analysis' or 'gene- based collapsing approach' or 'variant collapse analysis'.

**Response:**

- Thank you for your kind comments. Following your suggestion, we have changed the header from "Three VV genes identified by collapse analysis" to “*Three VV genes identified by rare variant collapsing analysis*”.

Whenever the authors write about 'significant level' it should probably be 'significance level', e.g.

L153 - The significant level of the genetic variation associations...

**Response:**

- Thank you for your kind comments. We have corrected the expression "significant level" in the manuscript to "significance level", as follows “*The significance level of the genetic variant associations*” and “*...with different significance levels across sexes...*”.

Table 2 - Put definition of 'rare' and 'ultra-rare' variants in Table 2.

**Response:**

- Thank you for your kind comments. We apologize for causing misunderstanding among reviewers. The definitions of rare variant and ultra-rare variant in our study are not the same as those in SAIGE-GENE+ (<https://saigegit.github.io/SAIGE-doc/docs/set_step2.html>). Rare variants in our study are defined as those with MAF < 0.01, and the number of rare variants in SAIGE-GENE+ and **Table 2** refers to the number of markers that are not ultra-rare with minor allele count in the set (MAC) > 10. The data presented in **Table 2** are the original results in SAIGE-GENE+, where ultra-rare refers to the default setting and is defined as MAC ≤ 10. We have apologetically generalized the number of rare variants in **Table 2** to apply to the definition of rare in our study and placed the definition of rare variants at the bottom of **Table 2** (“*Number of rare variants: number of markers that MAF < 0.01*”).

Should be clumping instead of clump

L214 - After clump, we identified ...

**Response:**

- Thank you for your kind comments. We have changed the expression “After clump, we identified...” to “*After clumping, we identified...*” in the **Manuscript**, and the expression of age-matched analysis in line 214 was removed due to the similar age distribution in the VV case and control groups based on another reviewer’ comment.

Does it mean that ECE1 was not associated in the age-matched analysis any longer?

L217 - Collapse analyses were also performed in the matched population and showed that population and showed that PIEZO1 and FBLN7 were still found to be correlated...

**Response:**

- Thank you for your kind comments. We did not find a significant association between *ECE1* and VV in the age-matched population, which may be related to population differences resulting from the exclusion of approximately 110,000 participants after matching. We have removed the age-matched analysis due to the similar age distributions of the VV case and control groups based on another reviewer's comment.

Remove 'And' in the following sentence.

L235 - And there were 12 GO terms significantly...

**Response:**

- Thank you for your kind comments. We have removed “And” in the sentence “And there were 12 GO terms significantly...” in **Results - Enrichment analysis of VV genes and their expression**.

Correct reference should be

L280 - Van Hout et al. study

**Response:**

- Thank you for your kind comments. We have changed the reference citation to “*Compared to the studies by Van Hout et al. and Wang et al., our study has a larger sample size and more validation of the findings (replication in the FinnGen cohort) and exploration of biological function (e.g. functional enrichment analysis, tissue expression analysis, and single-cell RNA sequencing analysis)*” in **Discussion**.

Maybe better phrase like this.

L308 - The other major finding in this study were the associations of VV with rare variants in three genes (PIEZO1, ECE1, and FBLN7) identified by the variant collapsing analysis.

**Response:**

- Thank you for your kind comments. Following your suggestion, we have changed the ambiguous expression in **Discussion** to “*The other findings in this study were the associations of VV with three rare variant genes (PIEZO1, ECE1, and FBLN7) identified by the gene-based collapsing analysis*”.

Probably better singular.

L320 - A mouse model showed that estrogen...

**Response:**

- Thank you for your kind comments. We have changed “A mice model showed that estrogen...” to “*A mouse model showed that estrogen...*” in **Discussion**.

Do the authors mean there were no other cohorts available with VV and WES data?

L363 - Previous WES of VV was lacking...

**Response:**

- Thank you for your kind comments. We apologize for the misunderstanding caused by our misrepresentation, and we have changed this ambiguous statement in **Discussion** to "*Due to the difficulty in obtaining exome sequencing data for VV from other databases, we validated our findings using summary statistics from FinnGen*".

Re-phrase the sentence in L367-369.

**Response:**

- Thank you for your kind comments. Based on your suggestion, we have changed the sentence "Then, we mainly referred to Ahmed et al. for defining VV phenotypes. The phenotype definition they used replicates well the loci of common variants when performing GWAS" to "*We defined the VV phenotype mainly based on the study of Ahmed et al., which successfully replicated most of the VV-related risk loci identified in previous studies using this definition*" in **Discussion**.

The cut-offs are missing here.

L421 - Sample-level QC included the removal of samples that were Ti/Tv, were Het/Hom and SNV/indel, had withdrawn their consent, ...

**Response:**

- Thank you for your kind comments. We have added the cut-offs in **Methods** and changed the sentence to “*Sample-level QC involved removing samples with Ti/Tv, Het/Hom, SNV/indel, number of singletons exceeding the mean ± 8SD, and discrepancies between self-reported and genetically inferred sex, as well as duplicates and withdrawn consent*”.

Maybe better 'related' than 'irrelevant'.

L426 - The kinship coefficient threshold of 0.0884 was used to classify secondary kinship as an irrelevant sample.

**Response:**

- Thank you for your kind comments. Following your suggestion, we have changed the expression “The kinship coefficient threshold of 0.0884 was used to classify secondary kinship as an irrelevant sample” to “*The kinship coefficient threshold of 0.0884 (the 2nd relatedness) was used to define a related sample*” in **Methods**.

Maybe better 'higher' than 'more significant'

L428 - kinship coefficients more significant than 0.0884

**Response:**

- Thank you for your kind comments. Following your suggestion, we have changed the expression “...kinship coefficients more significant than 0.0884...” to “*For pairs with kinship coefficients higher than 0.0884, we removed samples associated with multiple other individuals iteratively until none remained to maximize the sample size*” in **Methods**.

What procedure was actually used to calculate kinship?

**Response:**

- Thank you for your kind comments. We have provided the procedure actually used to calculate kinship in **Methods**: “*The kinship coefficient threshold of 0.0884 (the 2nd relatedness) was used to define a related sample. For pairs with kinship coefficients higher than 0.0884, we removed samples associated with multiple other individuals iteratively until none remained to maximize the sample size. Then we randomly removed one from the remaining pairs*”.

Which cut-offs were used?

L410-413 - A missense variant was considered damaging when it was predicted to be deleterious by SIFT (sorting intolerant from tolerant)(57), PolyPhen-2 HDIV, PolyPhen-2 HVAR(58), LRT (likelihood ratio test)(59) and MutationTaster(60) consistently...

**Response:**

- Thank you for your kind comments. We have added the description of the cut-off values for defining deleterious variants in each software in **Methods** (“*The definitions of deleterious variants for each software are given in* ***Additional file 3 (Supplementary Methods)***”) and **Additional file 3 - Supplementary Methods** (“*The cut-off used in SIFT: Positions with normalized probabilities less than 0.05 are predicted to be deleterious, and those greater than or equal to 0.05 are predicted to be tolerated. The cut-off used in PolyPhen-2: Current version 2.2 of the PolyPhen-2 uses 5%/10% false positive rate (FPR) for HumDiv model and 10%/20% FPR for HumVar model as the thresholds for this ternary classification. Mutations with their posterior probability scores associated with estimated false positive rates at or below the first (lower) FPR value are predicted to be probably damaging (more confident prediction). Mutations with the posterior probabilities associated with false positive rates at or below the second (higher) FPR value are predicted to be possibly damaging (less confident prediction). Mutations with estimated false positive rates above the second (higher) FPR value are classified as benign. The cut-off used in LRT (Likelihood Ratio Test): Deleterious mutations were predicted by nonsynonymous SNPs that disrupt significantly constrained codons defined by the LRT (P < 0.001) and several subsequent filters. The cut-off used in MutationTaster: If a variant is marked as probable-pathogenic or pathogenic in ClinVar, it is automatically predicted to be disease-causing, i.e. disease-causing automatic (the naive Bayes classifier is run nevertheless and the p-value for the prediction is shown)*”).
