# Supplementary material for: Exome sequencing identifies novel genetic variants associated with varicose veins
Source: PLoS Genet. 2024 Jul 9;20(7):e1011339. doi: 10.1371/journal.pgen.1011339 (PMC11233024; doi:10.1371/journal.pgen.1011339)
Supplement: Attachment — Submitted filename: Response to Reviewers.docx [file pgen.1011339.s031.docx]

The authors sincerely appreciate the critical reviews of the paper, and for the helpful way in which the reviewing editors put together a constructive list of suggestions for the revision of the paper. We have now revised the paper to carefully address all the points raised. Our responses below are preceded by “-”, and changes made to the paper are shown below within “…”, and in red font in the revised paper.

**Detailed Response to Editor**

- Many thanks for your positive feedback and for your high concern towards our manuscript. The reviewers' valuable comments indeed provided important guidance to our research. By thoughtfully integrating their insights, we revised the paper and believe the manuscript's quality has been improved.

**Detailed Response to Reviewers**

**Response to Reviewer #2**

Reviewer #2

The authors have addressed all the comments and suggested modifications. I have a few minor suggestions regarding wording before publication:

- In Table 2 legend: 'Number of rare variants: number of markers with MAF < 0.01.'

**Response:**

- Thank you for your kind comments. Based on your suggestion, we have changed our incorrect expression to “*Number of rare variants: number of markers with MAF < 0.01*” in **Table 2** legend.

- l. 189 and 484: 'defined as having a MAF between 1 × 10^-5^ and 1 × 10^-2^'

**Response:**

- Thank you for your kind comments. Based on your suggestion, we have changed our expression to “*defined as having a MAF between 1 × 10^-5^ and 1 × 10^-2^*” in **Line 189** and “*and MAF between 1 × 10^-5^ and 1 × 10^-2^*” in **Line 484**.

- l. 200: 'of the variant chr1:21227980:A:G'

**Response:**

- Thank you for your kind comments. Following your suggestion, we have corrected our expression in **Line 200** to “*of the variant chr1:21227980:A:G*”.

- l. 214 and methods: can the authors provide the numbers of males and females analyzed?

**Response:**

- Thank you for your kind comments. Following your suggestion, we have provided the number of males and females in **Line 214** (“*These analyses included 162,210 males and 188,560 females*”) and **Methods - Sensitivity and leave-one-variant-out analyses** (“*There were 162,210 participants in the male group and 188,560 in the female group after stratification by sex*”).

**Response to Reviewer #3**

Reviewer #3

The revised manuscript is much improved, however I still have a few minor comments.

The following expressions should be re-phrased because their use of English or their content do not sound quite correct:

L29. 'a lack of meticulous study on its genetic factors'

**Response:**

- Thank you for your kind comments. Following your suggestion, we have corrected our expression in **Line 29** to “*Varicose veins (VV) are one of the common human diseases, but the role of genetics in its development is not fully understood*”.

L39. 'The pleiotropic of these genes'

**Response:**

- Thank you for your kind comments. Following your suggestion, we have corrected “*The pleiotropic of these genes...*” in **Line 39** to “*The pleiotropic effects of VV-related genes...*”.

L235. 'However, no enrichment results were found due to fewer identified VV genes in males.'

**Response:**

- Thank you for your kind comments. Based on your suggestion, we have changed our expression to “*However, the VV genes identified in males were not successfully enriched for the same biological processes*” in **Line 235**.

L281. 'be help for therapeutic target discovery'

**Response:**

- Thank you for your kind comments. Based on your suggestion, we have changed our incorrect expression in **Line 281** to “...*be useful for therapeutic target discovery...*”.

L291. 'the significance of adipose in the association'

**Response:**

- Thank you for your kind comments. Based on your suggestion, we have corrected our incorrect expression in **Line 291** to “...*the importance of adipose tissue in the association...*”.

L355. 'association between overlapping gene and VV'

**Response:**

- Thank you for your kind comments. Based on your suggestion, we have changed our expression in **Line 355** to “...*the association between overlapping genes and VV risk*”.

L408. 'whole-exome sequencing on for all participants from the UKB'

**Response:**

- Thank you for your kind comments. Based on your suggestion, we have changed the incorrect expression in **Line 408** to “...*whole-exome sequencing on all participants from the UKB*”.

I am not clear what is meant by the following statement.

L426. Then we used KING software to calculate pairwise heterozygote concordance rates.

**Response:**

- Thank you for your kind comments. We have changed this expression in **Line 426** to “*KING(1) software was applied to calculate pairwise heterozygote concordance rates for each pair of samples and the kinship coefficient using high-quality variants*”. We mainly rely on the study of Jurgens et al.(2) who mention it in their Supplementary Methods: “We first used KING(1) (version 2.2.5) to calculate pairwise heterozygote concordance rates for each pair of samples, using the high-quality independent autosomal markers. Then we used the high-quality autosomal variants present in both WES and array datasets to compute per-sample heterozygote concordance rates between WES calls and genotyping array calls. We inferred the genetic sex of each participant with the --*check*-*sex* option in PLINK using the high-quality independent X-chromosomal markers. We set any sample with F > 0.8 to male, while samples with F < 0.5 were set to female. Finally, using all ~12.7M autosomal WES variants, we computed a number of additional metrics including sample call rate, transition/transversion ratio (Ti/v), heterozygote/homozygote ratio (Het/Hom), SNV/indel ratio (SNV/indel) and the number of singletons”.
- **Reference**:

1.	Manichaikul A, Mychaleckyj JC, Rich SS, Daly K, Sale M, Chen WM. Robust relationship inference in genome-wide association studies. Bioinformatics (Oxford, England). 2010;26(22):2867-73.

2.	Jurgens SJ, Choi SH, Morrill VN, Chaffin M, Pirruccello JP, Halford JL, et al. Analysis of rare genetic variation underlying cardiometabolic diseases and traits among 200,000 individuals in the UK Biobank. Nature genetics. 2022;54(3):240-50.

**Reference**

1.	Manichaikul A, Mychaleckyj JC, Rich SS, Daly K, Sale M, Chen WM. Robust relationship inference in genome-wide association studies. Bioinformatics (Oxford, England). 2010;26(22):2867-73.

2.	Jurgens SJ, Choi SH, Morrill VN, Chaffin M, Pirruccello JP, Halford JL, et al. Analysis of rare genetic variation underlying cardiometabolic diseases and traits among 200,000 individuals in the UK Biobank. Nature genetics. 2022;54(3):240-50.
